# Mini-AUV Hydrodynamic Parameters Identification via CFD Simulations and Their Application on Control Performance Evaluation

**DOI:** 10.3390/s21030820

**Published:** 2021-01-26

**Authors:** José J. Castillo-Zamora, Karla A. Camarillo-Gómez, Gerardo I. Pérez-Soto, Juvenal Rodríguez-Reséndiz, Luis A. Morales-Hernández

**Affiliations:** 1 L2S of Université Paris Sud-CNRS-CentraleSupelec, Université Paris Saclay, 91190 Gif-sur-Yvette, France; jose.castillo@ipsa.fr; 2 IPSA Paris, 94200 Ivry-sur-Seine, France; 3 Mechanical Engineering Department, Tecnológico Nacional de México/Instituto Tecnológico de Celaya, Celaya, Guanajuato 38010, Mexico; 4 Faculty of Engineering, Universidad Autónoma de Querétaro, Santiago de Querétaro, Querétaro 76010, Mexico; israel.perez@uaq.mx (G.I.P.-S.); juvenal@uaq.edu.mx (J.R.-R.); 5 Faculty of Engineering, Universidad Autónoma de Querétaro, San Juan del Río, Querétaro 76807, Mexico; luis.morales@uaq.mx

**Keywords:** mini-AUV, hydrodynamic parameters identification, CFD simulation, ANSYS™, position controllers

## Abstract

This manuscript presents a fully detailed methodology in order to identify the hydrodynamic parameters of a mini autonomous underwater vehicle (mini-AUV) and evaluate its performance using different controllers. The methodology consists of close-to-reality simulation using a Computed Fluid Dynamics (CFD) module of the ANSYS™ Workbench software, the processing of the data, obtained by simulation, with a set of Savistky–Golay filters; and, the application of the Least Square Method in order to estimate the hydrodynamic parameters of the mini-AUV. Finally, these parameters are considered to design the three different controllers that are based on the robot manipulators theory. Numerical simulations are carried out to evaluate the performance of the controllers.

## 1. Introduction

The increasing implementation of underwater vehicles in ocean exploration for scientific or commercial benefits, rescue maneuvers, and transporting operations implies an efficiency improvement in matters of design, as well as the development of new control techniques and methodologies, which, in conjunction, evoke significant advances in terms of technology for underwater applications [1,2,3].

The identification of the hydrodynamic parameters of autonomous underwater vehicles (AUVs) at the early stages of conception stands as the main motivation of the present work, since the hydrodynamic parameters could potentially serve as a measure of the design efficiency [4,5,6,7].

In order to estimate the hydrodynamic parameters of a prescribed underwater vehicle, different experimental methodologies have been reported in the literature [4,5,6,7,8,9,10,11,12,13,14,15,16,17], yet, the majority of them require the implementation of top-technological expensive gadgets and tools, which makes the design task difficult for those researchers whose budget and facilities are an issue to overcome. In this regard, the implementation of Computed Fluid Dynamics (CFD) software and other computational resources has emerged as an alternative to conceive the vehicles at the first stages of research.

Recent publications provide evidence of the efforts to improve hydrodynamic parameters estimation techniques, emphasizing the increasing importance of the implementation of computational resources. In [18], a maximum likelihood identification algorithm in conjunction with CFD calculations is proposed and validated via experimentation. On the other hand, the hydrodynamic parameters are treated as a function of the angular position in the performance of sharp maneuvers, as exposed in [19,20]. A control-oriented modeling approach is proposed for a low-speed complex-shaped semi-AUV at [21], and a low-cost, efficient CFD procedure is introduced to compute the parameters.

The methodologies that were adopted by a scientist to accurately estimate the vehicles hydrodynamic characteristics are mainly those that have been used in experimental studies; thus, the issue to overcome, besides the computational cost, is to imitate the real scenario conditions in the software environment; issue that has been widely discussed in the literature and that has gathered the attention of researchers in early decades [6,7,8,9,10,17].

The authors consider that the efforts for modeling the hydrodynamic effects in a virtual environment have produced a faster conception and evaluation processes of the vehicles, as such, an action facilitates the recursive improvement process, similar to that of the control algorithms validation [1,2,3,22].

In the control vein, the knowledge of the hydrodynamics coefficients and the physical properties of the system could lead to a potential misconception of the control laws, whereas they are used for position stabilization [23] or trajectory tracking and path planning [22], among other complex tasks [24,25,26]. Nonetheless, the estimation techniques and observers theory have been adopted in maritime engineering, since they allow for the vehicle to know the external disturbances and the hydrodynamic effects that deteriorate the system itself, extending the concept of “Autonomous” vehicles.

At the early stages of the design of underwater vehicles, the stability of the system is a concern that draws the attention of researchers, since the success of the majority of other maneuvers highly depends on the capability of the robot to stay at a given desired point, and to remain at a specific orientation, as could be the study of sunken ships, species observation, and the monitoring of underwater structures, to mention a few [1,2,3].

A brief survey on this topic gives evidence of the diverse techniques that were implemented to ensure the stability of underwater vehicles [23,24,25,26], being the PD and PID controllers, with different variations, the most commonly adopted. The aforementioned controllers have been implemented in order to accomplish tasks as depth control under continuous waves conditions [27,28,29,30] or the path following adversarial environmental conditions [31]. Nonetheless, robust control techniques have been adopted in order to improve the behavior of the system, as in [32,33,34], where the corresponding authors show the effectiveness of the Sliding Mode Control (SMC) applied over these vehicles, extending the capabilities of the theory to the observation domain [35]. Moreover, some techniques [36] consider an iterative identification method to estimate the parameters of the system and the design of the controller at the same time.

In all of the cited works, the control design considers that the physical properties and the hydrodynamic coefficients of the system are known, even when some observers are implemented to compute such coefficients on-board; these are needed before the real operation takes place, as the estimations must be validated. Thus, characterizing the vehicle in terms of the hydrodynamics has a considerable impact on the overall performance of the AUVs.

### Problem Statement and Paper Contributions

The early identification of the hydrodynamic effects that a mini-AUV experiences during motion is the issue to overcome in this manuscript by:

1. The adaptation of a yet-virtually-translated experimental test set [11,12,13] to the environment of the CFX on ANSYS™ Workbench software (ANSYS CFX) may be referred to as the industry-leading computational fluid dynamics software for turbo-machinery applications.
2. The simulation of the mini-AUV as a submerged body in the ANSYS CFX environment for determining the forces, accelerations, and velocities during motion.
3. The conception of the control laws and the stability study, including the estimated hydrodynamic parameters of the mini-AUV, through the robot manipulator control theory, in order to validate the behavior of the system.

It must be highlighted that, even when Finite Volume Method (FVM) simulations are most commonly adopted for hydrodynamics and fluid applications, this work considers, at the same time, the FEM simulation, as it is more appropriate for structural applications, which include forces definition. The ANSYS CFX module manages both methods, which allows for an accurate simulation of both phenomena [37,38]. Nonetheless, recent works on the literature report, for specific cases of study and simulation conditions, slight differences in computation efficiency between the methods mentioned above, and the results do not vary significantly [39,40].

In this regard, the current manuscript is structured by six sections and an appendix; the description of the mini-AUV, including its dynamics, is presented in Section 2. Section 3 establishes the procedure in order to estimate the corresponding hydrodynamic parameters and the conditions programmed within the software environment. In Section 4, the conception of the controllers and corresponding Lyapunov stability analysis are presented, while Section 5 presents the results of the hydrodynamic parameters identification process and the position control task. The concluding remarks and the proposal of future work are comprised in Section 6. Finally, Appendix A contains all of the data generated during the computing process of the hydrodynamic parameters that are exposed in Section 3.

## 2. Mini-AUV Mathematical Modelling

**Figure 1:**
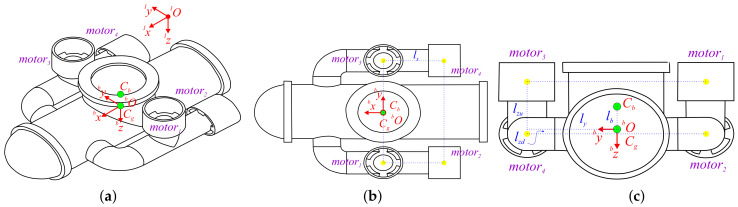
Mini-autonomous underwater vehicles (AUV): (**a**) Isometric view. (**b**) Top view. (**c**) Frontal view.

The current work considers the mini-AUV prototype that was conceived at the Institute and is depicted in Figure 1, in which the four propellers propulsion system is easily identified. Furthermore, the inertial reference frame ${}^{I}O_{{}^{I}x^{I}y^{I}z}$ and the body–fixed reference frame ${}^{b}O_{{}^{b}x^{b}y^{b}z}$ are defined. It is worth highlighting that the origin of the body–fixed reference frame coincides with the center of gravity $C_{g}$ of the mini-AUV. $C_{b}$ stands for the buoyancy center of the vehicle, which is supposed to be aligned with $C_{g}$.

The maximal dimensions of the vehicle (established as 0.6×0.42×0.24 m in ${}^{b}x$, ${}^{b}y$, and ${}^{b}z$, respectively) place it in the “mini vehicle” category [23,41]. According to the CAD software that was used for its conception (Solidworks™), the volume of the mini-AUV is 0.006 m${}^{3}$, which in conjunction with its mass, m=7.5 kg, defines the principal moments of inertia about the axis ${}^{b}x$, ${}^{b}y$, and ${}^{b}z$ as $I_{x}=0.11\;{kg\cdot m}^{2}$, $I_{y}=0.199\;{kg\cdot m}^{2}$, and $I_{z}=0.251\;{kg\cdot m}^{2}$, respectively.

The additional information that is provided by Figure 1b,c regarding the position of the motors, with respect to the body–fixed reference frame, is used in the following paragraphs to define the dynamics of the mini-AUV.

According to the Newton–Euler method [42,43], the dynamics of a rigid body of mass m>0∈IR with six-Degrees of Freedom (DOFs), and whose principal axis of inertia (with their respective moments of inertia $I_{x},\;I_{y},\;I_{z}>0\in I\;R$) coincide with the axis of the body–fixed reference frame, and the corresponding origin matches with the center of gravity, as in the case of the mini-AUV, is described by:

(1) $$M_{rb}{\dot{q}}_{rb}+C_{rb}q_{rb}+G_{rb}=\tau_{rb}$$

such that:

(2) $$\begin{array}{c} q_{rb}=\left[\begin{array}{c} \dot{\xi }\\ \omega \end{array}\right],\;G_{rb}=\left[\begin{array}{c} mg\\ 0_{3} \end{array}\right],\;\tau_{rb}=\left[\begin{array}{c} F_{t}\\ \tau_{t} \end{array}\right]\in {I\;R}^{6};\;M_{rb}=\left[\begin{array}{cc} mI_{3} & 0_{3}\\ 0_{3} & \tilde{I} \end{array}\right],\;C_{rb}=\left[\begin{array}{cc} 0_{3} & 0_{3}\\ 0_{3} & S\left(\omega \right)\tilde{I} \end{array}\right]\in {I\;R}^{6\times 6} \end{array}$$

where $\xi ={\left[x\;y\;z\right]}^{T}\in {I\;R}^{3}$, and $\omega ={\left[p\;q\;r\right]}^{T}\in {I\;R}^{3}$ denote the position of the vehicle in the space and the rotational velocities in the body–fixed reference frame, respectively, $g={\left[0\;0\;-g\right]}^{T}\in {I\;R}^{3}$ stands for the gravity vector, $0_{3}\in {I\;R}^{3}$ does for the zero vector, $I_{3}\in {I\;R}^{3\times 3}$ and $0_{3}\in {I\;R}^{3\times 3}$ stand for the identity and the zero matrices, $\tilde{I}=diag\left(I_{x},I_{y},I_{z}\right)\in {I\;R}^{3\times 3}$ does for the rotational inertia tensor, and $S\left(★\right)$ is the skew-symmetric operator over the vector $★\in {I\;R}^{3}$. In this regard, $F_{t}$ and $\tau_{t}$ include, correspondingly, the forces and torques that are produced by the propulsion system $f_{p},\tau_{p}\in {I\;R}^{3}$, and the hydrodynamic effects due to the motion of the vehicle $f_{h},\tau_{h}\in {I\;R}^{3}$, not to mention the influence of the external disturbances, which is neglected in this paper. In this sense:

(3) $$\tau_{rb}=\left[\begin{array}{c} F_{t}\\ \tau_{t} \end{array}\right]=\left[\begin{array}{c} f_{p}\\ \tau_{p} \end{array}\right]+\left[\begin{array}{c} f_{h}\\ \tau_{h} \end{array}\right]$$

Taking the geometry of the mini-AUV depicted in Figure 1, and the forces produced by each motor $f_{i}\in I\;R$ (with i=1,2,3,4), into consideration, it leads to:

(4) $$\begin{array}{c} f_{p}=\left[\begin{array}{c} f_{x}\\ f_{y}\\ f_{z} \end{array}\right]=\left[\begin{array}{ccc} C_{\theta }C_{\psi } & S_{\phi }S_{\theta }C_{\psi }-C_{\phi }S_{\psi } & C_{\phi }S_{\theta }C_{\psi }+S_{\phi }S_{\psi }\\ C_{\theta }S_{\psi } & S_{\phi }S_{\theta }S_{\psi }+C_{\phi }C_{\psi } & C_{\phi }S_{\theta }S_{\psi }-S_{\phi }C_{\psi }\\ -S_{\theta } & C_{\theta }S_{\phi } & C_{\theta }C_{\phi } \end{array}\right]\left[\begin{array}{c} f_{2}+f_{4}\\ 0\\ f_{1}+f_{3} \end{array}\right]=R\left[\begin{array}{c} f_{2}+f_{4}\\ 0\\ f_{1}+f_{3} \end{array}\right] \end{array}$$

(5) $$\begin{array}{c} \tau_{p}=\left[\begin{array}{c} \tau_{p}\\ \tau_{q}\\ \tau_{r} \end{array}\right]=\left[\begin{array}{c} l_{y}\left(f_{3}-f_{1}\right)+d\left(f_{2}+f_{4}\right)\\ l_{zd}\left(f_{2}+f_{4}\right)\\ l_{y}\left(f_{2}-f_{4}\right)+d\left(f_{1}+f_{3}\right) \end{array}\right] \end{array}$$

where $R\in {I\;R}^{3\times 3}$ provides a vector mapping from to the body–reference frame to the inertial frame and it corresponds to the rotation matrix that is described by the Euler angles contained in the vector $\eta ={\left[\phi \;\theta \;\psi \right]}^{T}\in {I\;R}^{3}$ whose rate of change, $\dot{\eta }\in {I\;R}^{3}$, is related to ω, such that:

(6) $$\omega =W\dot{\eta }\;with\;W=\left[\begin{array}{ccc} 1 & 0 & -S_{\theta }\\ 0 & C_{\phi } & S_{\phi }C_{\theta }\\ 0 & -S_{\phi } & C_{\phi }C_{\theta } \end{array}\right]\in {I\;R}^{3\times 3}$$

Additionally, d∈IR stands for the proportional factor that relates the force that is produced by the propeller to its corresponding exerted free moment $\tau_{i}=df_{i}\in I\;R$.

On the other hand, the hydrodynamic forces and torques, $f_{h}\;and\;\tau_{h}\in {I\;R}^{3}$, respectively, are produced by complex phenomena, which include the added mass effects, radiation-induced potential damping, and restoring forces. The magnitude of these terms depends on the velocities of the body expressed in the body–reference frame $\upsilon =R^{T}\dot{\xi }$ and ω; thus, they are provided in the reference frame that is mentioned above and modeled by an equation of the form of Equation (1) with an added mass inertia matrix $M_{a}>0\in {I\;R}^{6\times 6}$, a Coriolis terms matrix $C_{a}\in {I\;R}^{6\times 6}$, a damping matrix $D_{d}>0\in {I\;R}^{6\times 6}$, and a vector of restoring forces $G_{r}\in {I\;R}^{6}$, which comprises the buoyant force B∈IR. A fully extended treatment of these terms can be consulted at [11,12,13,44,45,46,47]. Furthermore, the SNAME notation, regarding the hydrodynamic parameters, is adopted now-on, and the hydrodynamic effects are included within the dynamics of the mini-AUV, such that Equation (1) can be rewritten as:

(7) $$M_{t}{\dot{q}}_{rb}+C_{t}q_{rb}+D_{d_{m}}q_{rb}+G_{t}=\left[\begin{array}{c} f_{p}\\ \tau_{p} \end{array}\right]$$

with $M_{t}=M_{rb}+M_{a_{m}},\;C_{t}=C_{rb}+C_{a_{m}},\;G_{t}=G_{rb}+G_{r_{m}}$. It must be highlighted that the matrices $M_{a_{m}},\;C_{a_{m}},\;D_{d_{m}}$ and the vector $G_{r_{m}}$ correspond, respectively, to $M_{a},\;C_{a},\;D_{d}$, and $G_{r}$ with the proper conversion of the translational terms to the inertial frame.

Because the mini-AUV dynamics is highly nonlinear and coupled, several assumptions are suggested to be considered throughout the literature [11,12,13,46,47]. In this regard, the off-diagonal elements of the matrix $M_{a}$ are neglected, as they are smaller than those of the diagonal; the mini-AUV is assumed to be naturally stable in roll and pitch ($\phi =\theta =0^{\circ }$ and p=q=0deg/s∀t≥0s), and $v=\dot{v}\approx 0$, as the actuators only produce forces along the body axis ${}^{b}x$ and ${}^{b}z$. Additionally, the mini-AUV operates at low speeds, such that the Coriolis and centripetal effects, and the quadratic damping terms, are small enough to be neglected yet, the factor $X_{u\left|u\right|}$ is still considered, since u>>w and u>>r; thus, the six-DOFs model of the mini-AUV can be reduced to a four-DOFs dynamical model of the form:

(8) $$\begin{array}{c} M_{q}\ddot{q}+C_{q}\dot{q}+D_{q}\dot{q}+G_{q}={\left[\begin{array}{cc} f_{p}^{T} & \tau_{r} \end{array}\right]}^{T} \end{array}$$

(9) $$\begin{array}{c} M_{\gamma }\dot{\gamma }+D_{\gamma }\gamma +G_{\gamma }=\tau_{\gamma } \end{array}$$

where Equation (8) holds for the motion that is described in the inertial frame, and Equation (9) does the proper for the motion described in the body reference frame. Additionally, $q={\left[x\;y\;z\;\psi \right]}^{T}\in {I\;R}^{4}$, $\gamma ={\left[u\;v\;w\;r\right]}^{T}$ and

(10) $$\begin{array}{c} M_{q}=JM_{\gamma }J^{T}\;;\;M_{\gamma }=diag\left(m+X_{\dot{u}},m+Y_{\dot{v}},m+Z_{\dot{w}},I_{z}+N_{\dot{r}}\right)\in {I\;R}^{4\times 4} \end{array}$$

(11) $$\begin{array}{c} C_{q}=\left[\begin{array}{cccc} -S_{\psi }C_{\psi }\left(Y_{\dot{v}}-X_{\dot{u}}\right) & -\left(S_{\psi }^{2}Y_{\dot{v}}+C_{\psi }^{2}X_{\dot{u}}\right) & 0 & 0\\ C_{\psi }^{2}Y_{\dot{v}}+S_{\psi }^{2}X_{\dot{u}} & S_{\psi }C_{\psi }\left(Y_{\dot{v}}-X_{\dot{u}}\right) & 0 & 0\\ 0 & 0 & 0 & 0\\ 0 & 0 & 0 & 0 \end{array}\right]\dot{\psi }\in {I\;R}^{4\times 4} \end{array}$$

(12) $$\begin{array}{c} D_{q}=JD_{\gamma }J^{T}\;;\;D_{\gamma }=diag\left(X_{u}+X_{u\left|u\right|}\left|u\right|,Y_{v},Z_{w},N_{r},\right)\in {I\;R}^{4\times 4}\; \end{array}$$

(13) $$\begin{array}{c} G_{q}={\left[\begin{array}{cccc} 0 & 0 & B-mg & 0 \end{array}\right]}^{T}\;;\;G_{\gamma }=J^{-1}G_{q}\in {I\;R}^{4} \end{array}$$

(14) $$\begin{array}{c} \tau_{\gamma }=J^{-1}{\left[\begin{array}{cc} {\left(f_{p}\right)}^{T} & \tau_{r} \end{array}\right]}^{T}={\left[\begin{array}{cccc} X & Y & Z & N \end{array}\right]}^{T}\in {I\;R}^{4} \end{array}$$

(15) $$\begin{array}{c} J=\left[\begin{array}{cccc} C_{\psi } & -S_{\psi } & 0 & 0\\ S_{\psi } & C_{\psi } & 0 & 0\\ 0 & 0 & 1 & 0\\ 0 & 0 & 0 & 1 \end{array}\right]\in {I\;R}^{4\times 4}\; \end{array}$$

Notice that the *v* dynamics has been considered to keep the matrices within ${I\;R}^{4\times 4}$.

Equations (8) and (9) are respectively used in the following sections to identify the hydrodynamic parameters of the mini-AUV, and to conceive the controllers and their stability analysis; meanwhile, Equation (7) was used to carry out the corresponding numerical simulations to validate the controllers.

## 3. Hydrodynamic Parameters Identification

The experimental methodology that was presented in [11,12,13,14,15] was considered to identify the hydrodynamic parameters of the vehicle. This methodology implies the study of the performance of the vehicle in only one DOF at a time. The vehicle is constrained on the other 5-DOFs.

The process to identify the parameters of the mini-AUV consists of finding the numeric values of the unknown constants of Equation (9). To this end, ANSYS CFX was used to simulate the real environmental conditions in order to obtain the forces acting over the mini-AUV and the corresponding velocities. The accelerations were computed using a Savitzky–Golay filter and the Least Squares Theory was implemented to treat the overall data [44,45].

### 3.1. Basis of the Least Squares Method

Through the basis of the Least Squares Theory, an estimation of unknown parameters can be obtained by following the set equations:

(16) $$\begin{array}{c} {\hat{\theta }}_{\mathrm{LS}}={\left(H^{T}H\right)}^{-1}H^{T}\vartheta \;;\;{\hat{\sigma }}_{\theta }=\sqrt{diag\left(cov\left({\hat{\theta }}_{\mathrm{LS}}\right)\right)}; \end{array}$$

(17) $$\begin{array}{c} \mathrm{cov}\left({\hat{\theta }}_{\mathrm{LS}}\right)={\hat{\sigma }}^{2}{\left(H^{T}H\right)}^{-1}\;;\;{\hat{\sigma }}^{2}=\frac{{\left(\vartheta -H{\hat{\theta }}_{\mathrm{LS}}\right)}^{T}\left(\vartheta -H{\hat{\theta }}_{\mathrm{LS}}\right)}{\dim\left(\vartheta \right)-\dim\left({\hat{\theta }}_{LS}\right)} \end{array}$$

where ${\hat{\theta }}_{\mathrm{LS}}$ is the vector of estimated parameters, *H* is the deterministic matrix model, ϑ the measurement vector (forces vector), ${\hat{\sigma }}_{\theta }$ is the variance, and $\mathrm{cov}\left({\hat{\theta }}_{\mathrm{LS}}\right)$ is the covariance; meanwhile, ${\hat{\sigma }}^{2}$ stands for the standard deviation of the studied data set [48,49].

The Equation (9) model can be extended in order to make the equations of motion satisfy the required format to apply the Least Squares Methodology. Thus, it follows, for the surge (${}^{b}x$) motion representative example, that:

(18) $$m_{x}\dot{u}+X_{u}u+X_{u\left|u\right|}\left|u\right|u=X$$

with the constant $m_{x}=m+X_{\dot{u}}$.Furthermore, the mini-AUV is observed to have no symmetry with respect to the ${}^{b}y{\;}^{b}z$ and ${}^{b}x{\;}^{b}y$ planes (Figure 1), which implies that the hydrodynamic parameters for the positive and negative motion must be estimated [11,44,45]. Notice that, in yaw motion, the parameters remain the same for turning right or left due to the symmetry of the vehicle in the ${}^{b}x{\;}^{b}z$ plane.

Equation (18) can be expressed in a matrix shape, for *n* data points, as:

(19) $$\left[\begin{array}{c} X_{1}\\ X_{2}\\ X_{3}\\ \vdots \\ X_{n} \end{array}\right]=H\left[\begin{array}{c} m_{x}\\ X_{\dot{u}}\\ X_{\left|u\right|u} \end{array}\right]\;\mathrm{with}\;H=\left[\begin{array}{ccc} {\dot{u}}_{1} & u_{1} & \left|u_{1}\right|u_{1}\\ {\dot{u}}_{2} & u_{2} & \left|u_{2}\right|u_{2}\\ {\dot{u}}_{3} & u_{3} & \left|u_{3}\right|u_{3}\\ \vdots & \vdots & \vdots \\ {\dot{u}}_{n} & u_{n} & \left|u_{n}\right|u_{n} \end{array}\right]\in {I\;R}^{n\times 3}$$

that fits to Equations (16) and (17) as ${\hat{\theta }}_{\mathrm{LS}}={\left[m_{x}\;X_{\dot{u}}\;X_{\left|u\right|u}\right]}^{T}$ and $\vartheta ={\left[X_{1}\;X_{2}\;X_{3}\;\cdots \;X_{n}\right]}^{T}$.

Next, the conditions that are considered in the software ANSYS CFX to perform the simulations are described.

### 3.2. ANSYS CFX Simulation Environment

**Figure 2:**
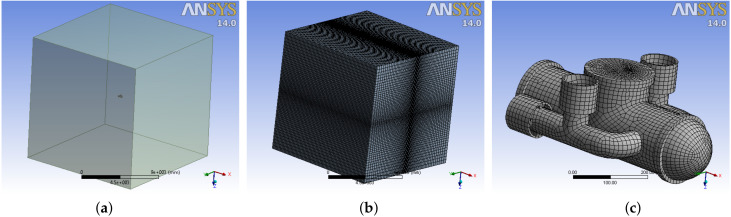
Mini-AUV in ANSYS CFX environment: (**a**) Vehicle and fluid domain. (**b**) Fluid mesh. (**c**) Vehicle mesh.

As a first step into the simulation software, the domains were defined, one for the fluid domain and the other for the rigid body, i.e., the mini-AUV. The control volume or fluid domain was established to be a cube with dimensions 15m×15m×15 m. Afterward, the CAD model of the mini-AUV was imported to the software and then placed at the center of the cube to ensure the fully submerged condition, as shown in Figure 2.

The *sweep method* and two different *sizings* were programmed to generate the fluid mesh. The *sizings* were applied to the edges of the cube to obtain more elements near the mini-AUV [8,10]. Both *hlsizings* were set to give 70 divisions on the edges in ${}^{I}y$ and ${}^{I}z$. The change rate, which defines the ratio size between the inner and the outer elements of the edge, was set to 5, obtaining 343,000 elements and 357,911 nodes with a mean element quality of 0.752476. Figure 2b depicts the result of this configuration.

Regarding the mesh of the mini-AUV, the *Hex dominant method* with *all quad* option was used. A sizing of 10 mm was also programmed with a change rate of 1.1 and a curve sensitivity of $25^{\circ }$, resulting in 32,708 elements and 25,428 nodes whose mean element quality was 0.751376. Figure 2c offers a visual reference of the mini-AUV mesh.

In total, 375,708 elements and 383,339 nodes were generated with a global mean quality of 0.751476, which coincides with the literature reports [8,9,10,37], and that allows to consider such a mesh to be valid to perform the simulation.

The simulation time is defined as 30 s with two different time steps of 0.2 and 0.25 s in order to obtain 150 and 120 data points, respectively, to have a rough approach to the time step influence.

**Figure 3:**
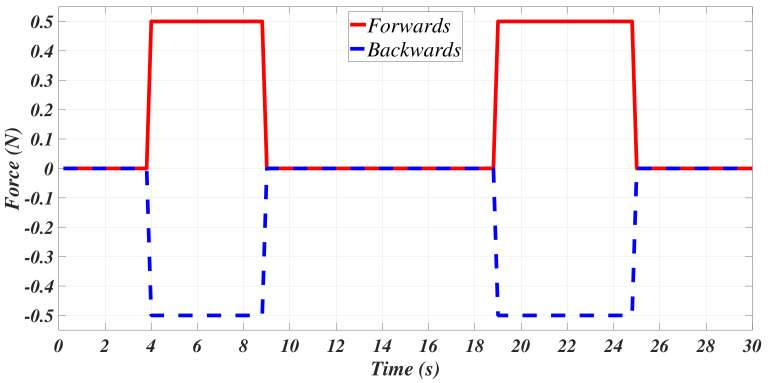
Programmed forces to generate the movement of the mini-AUV along ${}^{b}x$.

The default properties of the water available on ANSYS CFX were used to simulate the behavior of the mini-AUV in ${}^{b}x$ [37]. The buoyant effect was activated, while the mini-AUV was simulated as an immersed solid with the properties provided in Section 2. The scaling function was set to 100, as suggested in [37]. The submerged body option was also selected, allowing to apply the force, Figure 3, in order to produce the forward and backward motion of the mini-AUV.

Lastly, at the bottom and the sides of the fluid domain, the wall conditions were established; meanwhile, an opening was assigned at the top surface. The translational motion of the mini-AUV in ${}^{I}y$ and ${}^{I}z$ was constrained, as experimentally suggested by [11]. The results of the simulation and the data treatment are introduced and discussed in Section 5.1.

## 4. Controllers Conception

The controllers were designed with the mini-AUV hydrodynamic parameters when considering the control theory of robot manipulators exposed in [43]. For these ends, Equations (8) and (9) are cited, highlighting that the current analysis stands exclusively for the position control case, which implies that the desired point $q_{d}={\left[\begin{array}{cccc} x_{d} & y_{d} & z_{d} & \psi_{d} \end{array}\right]}^{T}\in {I\;R}^{4}$ does not vary with time, such that ${\dot{q}}_{d}={\ddot{q}}_{d}=0\in {I\;R}^{4}\;\forall \;t\geq 0$.

### 4.1. PD Controller

The PD control law with gravity compensation [43] can be adapted to the mini-AUV dynamics in Equation (8), such that:

(20) $$\tau_{q}=K_{P}\tilde{q}-K_{V}\dot{q}+G_{q}$$

where $\tilde{q}=q_{d}-q\in {I\;R}^{4}$ corresponds to the position error vector, $K_{P}>0\in {I\;R}^{4\times 4}$ stands for the proportional gains matrix, and $K_{V}>0\in {I\;R}^{4\times 4}$ does for the derivative gains matrix. The expression above can be related to $\tau_{\gamma }$ by the matrix *J*, such that $\tau_{\gamma }=J^{T}\tau_{q}$; thus, Equation (20) becomes:

(21) $$\tau_{\gamma }=J^{T}K_{P}\tilde{q}-J^{T}K_{V}J\gamma +G_{\gamma }$$

then, in conjunction with Equation (9), defines the closed-loop equation of the system as:

(22) $$M_{\gamma }\dot{\gamma }+D_{\gamma }\gamma +G_{\gamma }=J^{T}K_{P}\tilde{q}-J^{T}K_{V}J\gamma +G_{\gamma }$$

with the state of the system being defined as $x={\left[\begin{array}{cc} {\tilde{q}}^{T} & \gamma^{T} \end{array}\right]}^{T}\in {I\;R}^{8}$, and whose equilibrium point $x_{e}\in {I\;R}^{8}$ is located at ${\left[\begin{array}{cc} 0^{T} & 0^{T} \end{array}\right]}^{T}\in {I\;R}^{8}$ that can be computed by considering the closed-loop Equation (22) and solving:

(23) $$\dot{x}=\left[\begin{array}{c} -\dot{q}\\ \dot{\gamma } \end{array}\right]=\left[\begin{array}{c} -J\gamma \\ \dot{\gamma } \end{array}\right]=\left[\begin{array}{c} 0_{4}\\ 0_{4} \end{array}\right]$$

The stability of the system can be further addressed considering a Lyapunov candidate function of the form:

(24) $$V\left(\tilde{q},\gamma \right)=\frac{1}{2}\gamma^{T}M_{\gamma }\gamma +\frac{1}{2}{\tilde{q}}^{T}K_{P}\tilde{q}\geq 0$$

whose time derivative is given as:

(25) $$\dot{V}\left(\tilde{q},\gamma \right)=-\gamma^{T}J^{T}K_{V}J\gamma -\gamma^{T}D_{\gamma }\gamma$$

Accordingly, $\dot{V}\left(\tilde{q},\gamma \right)\leq 0\;\forall \;t\geq 0$, since $K_{V}$ and $D_{\gamma }$ are positive definite matrices by definition, thus the equilibrium is said to be stable [43].

Moreover, the LaSalle theorem leads to conclude that such stability is global asymptotic, since the set Ω is strictly defined, from Equations (22) and (25), as [42,43]:

$$\Omega =\left\{x:\dot{V}\left(\tilde{q},\gamma \right)=0\right\}=\left\{\tilde{q}=0\in {I\;R}^{4},\gamma =0\in {I\;R}^{4}\right\}$$

Following this vein, a PID controller conception, following the robot manipulators control theory, is introduced next.

### 4.2. PID Controller

The PID control law is proposed with a base on the theory available at [43]; in this regard, and compared with the PD controller, it includes a diagonal integral gain matrix $K_{I}>0\in {I\;R}^{4\times 4}$ such that:

(26) $$\tau_{q}=K_{P}\tilde{q}-K_{V}\dot{q}+K_{I}\int_{0}^{t}\tilde{q}\;dt+G_{q}$$

The integral action of the PID control law in Equation (26) introduces an additional state variable that is denoted herein by $\varphi \in {I\;R}^{4}$, and whose time derivative is $\dot{\varphi }=\tilde{q}\in {I\;R}^{4}$, leading to:

(27) $$\tau_{q}=K_{P}\tilde{q}-K_{V}\dot{q}+K_{I}\varphi +G_{q}$$

Equation (27) is related to the model in Equation (9), as explained in Section 4.1, in the sense that:

(28) $$M_{\gamma }\dot{\gamma }+D_{\gamma }\gamma +G_{\gamma }=J^{T}K_{P}\tilde{q}-J^{T}K_{V}J\gamma +J^{T}K_{I}\varphi +G_{\gamma }$$

To proceed with the stability analysis, the state vector, for this case of study, is defined, as follows:

(29) $$\chi ={\left[\begin{array}{ccc} \varphi^{T} & {\tilde{q}}^{T} & \gamma^{T} \end{array}\right]}^{T}\in {I\;R}^{12}$$

The only equilibrium point $\chi_{e}\in {I\;R}^{12}$ of the system matches the origin. Nevertheless, a Lyapunov stability analysis is conducted to prove it is asymptotically stable. In this regard, a global change of coordinates is executed to obtain a new state vector and its corresponding time derivative, respectively, as [42,43]:

(30) $$\chi^{*}=\left[\begin{array}{c} \varphi^{*}\\ \tilde{q}\\ \gamma \end{array}\right]=\left[\begin{array}{c} \iota \varphi +\tilde{q}\\ \tilde{q}\\ \gamma \end{array}\right]\in {I\;R}^{12}\;;\;{\dot{\chi }}^{*}=\left[\begin{array}{c} \iota \tilde{q}-J\gamma \\ -J\gamma \\ \dot{\gamma } \end{array}\right]\in {I\;R}^{12}$$

from where the closed–loop equation can be written as:

(31) $$M_{\gamma }\dot{\gamma }=J^{T}K_{P}\tilde{q}-\left(J^{T}K_{V}J+D_{\gamma }\right)\gamma +\frac{J^{T}K_{I}}{\iota }\left(\varphi^{*}-\tilde{q}\right)$$

with ι∈IR being a small positive constant.

Equation (31) results easy to be programmed due to the coordinates change as the integral term has been substituted, but the Lyapunov stability analysis is extensive. In this regard, Equation (28) can be translated to the inertial reference frame in order to develop the stability analysis; in this manner, the control law closes the loop, expressed as:

(32) $$M_{q}\ddot{q}+C_{q}\dot{q}+D_{q}\dot{q}+G_{q}=K_{P}\tilde{q}-K_{V}\dot{q}+K_{I}\varphi +G_{q}$$

With this representation of the system, a new vector of states is defined by $x^{*}\in {I\;R}^{12}$, such that:

(33) $$x^{*}={\left[\begin{array}{ccc} \kappa^{T} & q^{T} & \varphi^{T} \end{array}\right]}^{T}\in {I\;R}^{12}$$

where $\kappa =M_{q}\dot{q}\in {I\;R}^{4}$ is the generalized momentum of the mini-AUV [46]. Hence, it has been shown by the time differentiation of the Lyapunov candidate function of the form [43]:

$$V\left(x^{*}\right)={x^{*}}^{T}\left[\begin{array}{ccc} {M_{q}}^{-1} & \iota I_{4} & 0_{4}\\ \iota I_{4} & K_{P} & K_{I}\\ 0_{4} & K_{I} & \iota K_{I} \end{array}\right]x^{*}$$

with $I_{4}\in {I\;R}^{4\times 4}$ and $0_{4}\in {I\;R}^{4\times 4}$ standing for the identity and the zero matrices, respectively; that $\dot{V}\left(x^{*}\right)\leq 0$, and that q converges to $q_{d}$ if:

$$\begin{array}{c} \lambda_{\min}\left\{K_{V}\right\}>\lambda_{\max}\left\{M_{q}\right\}\;;\;\lambda_{\min}\left\{K_{I}\right\}>0\;;\;\lambda_{\min}\left\{K_{P}\right\}>\lambda_{\max}\left\{K_{V}+\frac{2}{\iota }K_{I}\right\} \end{array}$$

and ι is small enough, such that the following expression holds:

$$\frac{1}{2}\left(1-\iota \right)K_{P}-\iota M_{q}+\frac{\iota }{2}\sum_{i=1}^{4}{\tilde{q}}_{i}\frac{\partial M_{q}}{\partial q_{i}}>0$$

Notice that this solution only guarantees local stability in a limited region about the origin of Equation (33), [42,46,47]. A robust Sliding Mode Control approach is also proposed in order to carry out the mini-AUV behavior comparison.

### 4.3. Sliding Mode Controller

Based on the theory exposed in [43,50], and the results exposed in [42], the sliding surface vector $S\in {I\;R}^{4}$ is defined as:

(34) $$S=-J\gamma +\lambda_{m}\tilde{q}$$

with $\lambda_{m}\in {I\;R}^{4\times 4}$ being a diagonal matrix of gains $\lambda_{q_{i}}>0$.

The attractive sliding surfaces vector $\dot{S}\in {I\;R}^{4}$ is established as:

(35) $$\dot{S}=-K_{Z}\mathrm{sign}\left(S\right)$$

with $K_{Z}\in {I\;R}^{4\times 4}$ a matrix of gains $K_{z_{i}}>0$.

Equation (35) corresponds, at the same time, to the time derivative of Equation (34), which could also be defined as:

(36) $$\dot{S}=-\dot{J}\gamma -J\dot{\gamma }-\lambda_{m}J\gamma$$

Thus:

(37) $$K_{Z}\mathrm{sign}\left(S\right)=\dot{J}\gamma +J\dot{\gamma }+\lambda_{m}J\gamma$$

The stability analysis of this controller is developed according to the theory that was described in [43], which corresponds to robot manipulators control theory.

Let the state of the system be described by the vector S, from Equation (37), so that it can be concluded that the origin of the system is the only equilibrium point [42,43,50].

From the definitions of S and $\dot{S}$ in Equations (34) and (35), respectively, it holds that:

(38) $$S^{T}\dot{S}\leq 0$$

The Lyapunov candidate function is defined as:

(39) $$V\left(S\right)=\frac{1}{2}S^{T}S\geq 0$$

The time derivative corresponds to Equation (38), thus Equation (39) is a Lyapunov function, and the origin of the system is said to be stable [43].

The system shown in Equation (37) is autonomous; thus, the set Ω might be defined to apply the LaSalle theorem [43]:

(40) $$\Omega =\left\{S\in {I\;R}^{4}:\dot{V}\left(S\right)=0\right\}$$

It is straightforward to conclude, from Equation (38), that S=0 is the only initial condition in (40); thus, the stability of the origin is asymptotic [42,43,50].

The three controllers that are studied here are validated in the following section, in which the CFX simulation results are also included.

## 5. Simulation Results

In this section, the ANSYS CFX simulation results and the treatment of the data obtained are exposed. In the second subsection, the hydrodynamic parameters identification results are used in order to design the controllers and show their accuracy by numerical simulations.

### 5.1. ANSYS CFX Simulation Results and Data Treatment

**Figure 4:**
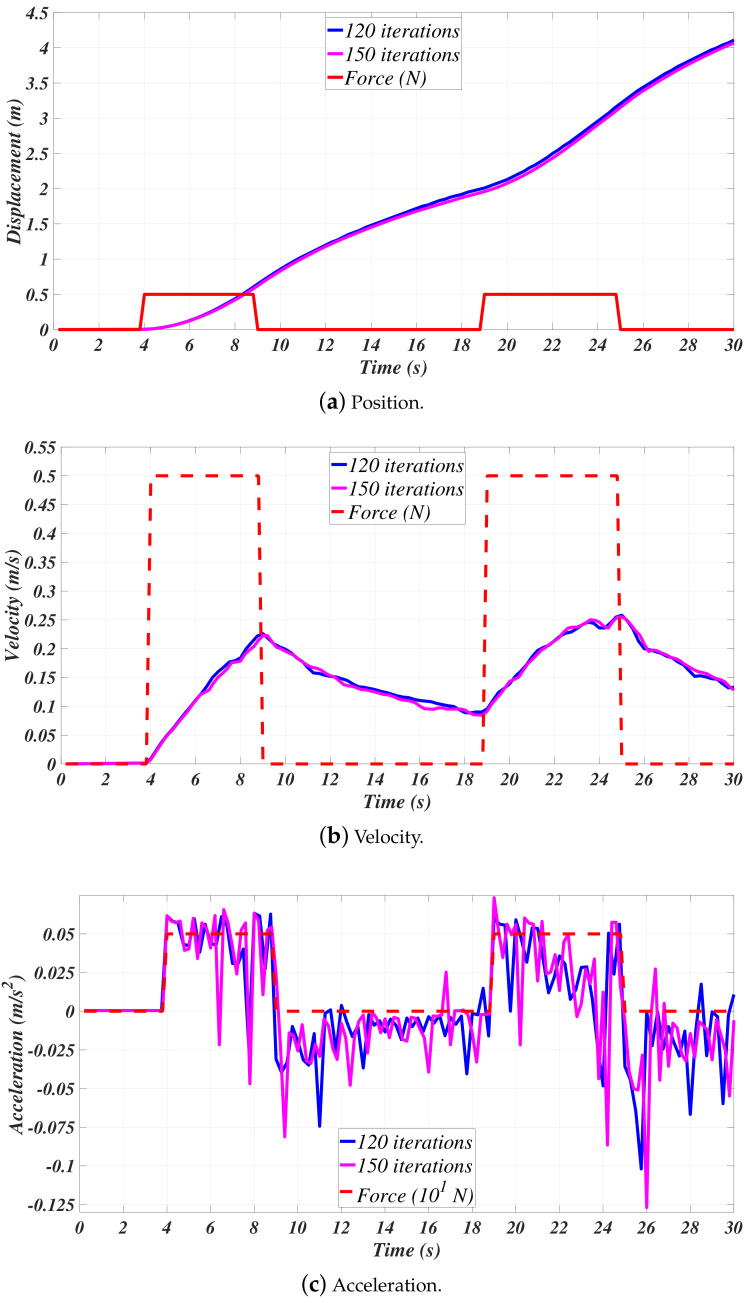
Surge (${}^{b}x$) motion.

**Figure 5:**
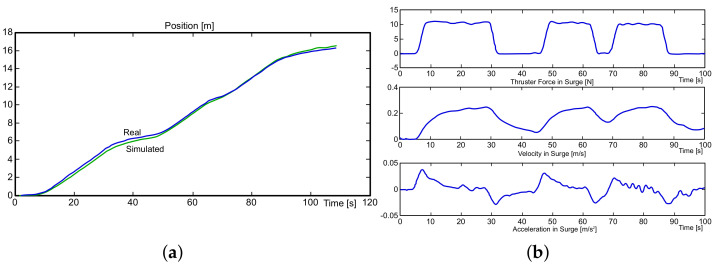
Experimental results of the GARBI vehicle concerning the positive surge motion: (**a**) Position. (**b**) Forces at the top, velocity in the middle, and acceleration at the bottom [11,12,13].

The information given by the fluid dynamics simulations was processed while using Matlab™. Figure 4 shows that the position, velocity, and acceleration of the mini-AUV, as provided by the ANSYS CFX module, are depicted, highlighting that such results correspond to its motion in $b^{x}$. Recalling [11,12,13,14,15,44,45], the plots can be considered to describe a close-to-reality behavior whose evidence is shown in Figure 5 [11,12,13] (Figures taken from [11,12,13] for comparison purposes only, no copyright infringement is intended.).

From Figure 4, it can be concluded that the time step variation of 0.05 s does not have a notorious impact on the displacement and velocity simulation results. On the other hand, notice that the position and velocities of the system follow the curves that were obtained by real experiments, with the differences due to the design and the dimensions of the vehicles being evident. Nevertheless, the accelerations seem not to have the same behavior, as the one provided by ANSYS CFX is noisy, in such a manner that it needs to be filtered, as suggested in the methodologies provided throughout [11,12,13,14,15]. In this vein, the acceleration results that are depicted in Figure 4 were used to estimate the parameters as an isolated possible scenario, as such behavior would potentially lead to a larger estimation error, as explained in the following paragraphs.

The Savitzky–Golay filter was used in order to filter the velocity and acceleration signals since, according to [11,51,52], the main filter characteristic relays on an accurate curve tracking and following, with an attenuation of abrupt signal changes.

In Matlab™, the function of the Savitzky–Golay filter is given by the instruction sgolayfilt(x,k,f). The input parameters of the function are: the vector *x*, which is the signal to be filtered, the polynomial grade *k*, and the size of the window f>k with this being an odd number [51,52].

The data were analyzed with a set of different filter parameters to evaluate the performance of the filter. Moreover, according to the documentation [51,52], the acceleration can also be computed from the velocity by applying the Savitzky–Golay method, which filters the input signal and numerically computes its first derivative. In Matlab™, this method is comprised of the function sgolay(N,F), where *N* is the polynomial grade, and *F* the size of the window. In Table 1, the parameters of the Savitzky–Golay filter and the corresponding case identifier number (label) are established.

**Figure 6:**
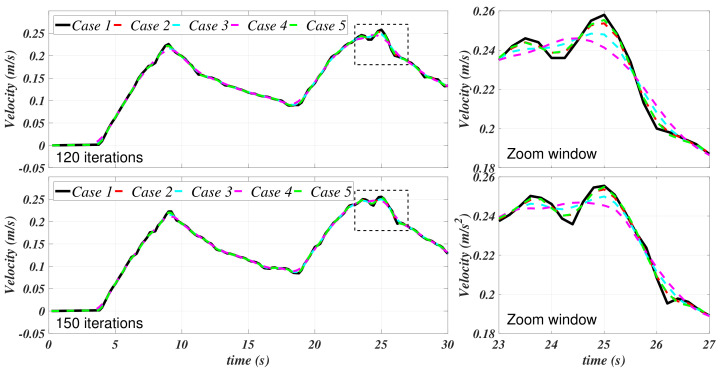
Filtered *u* for the filtering cases 1–5.

**Figure 7:**
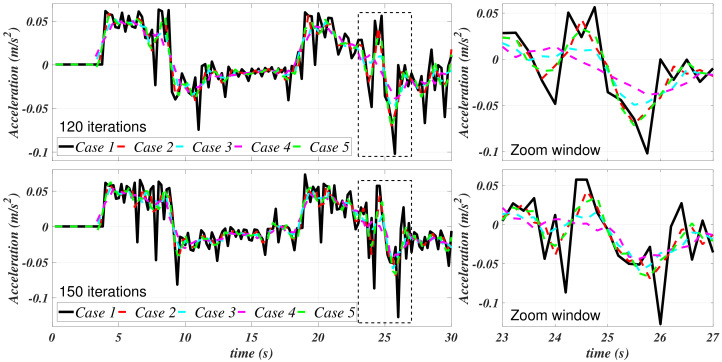
Filtered $\dot{u}$ for the filtering cases 1–5.

Case 1, as exposed in Table 1, corresponds to the data without any filtering process, i.e., as it was previously obtained from the ANSYS CFX simulation. Cases 2–5 stand for the cases in which a Savitzky–Golay filter was independently applied to the velocity and the acceleration. Figure 6 and Figure 7 depict the results of this filtering stage with the values that are provided in Table 1.

**Figure 8:**
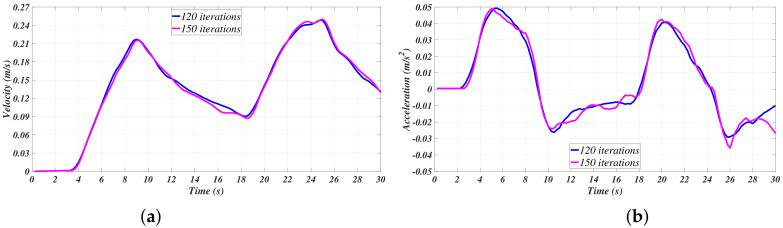
Case 3 comparison of filtered signals: (**a**) Velocity. (**b**) Acceleration.

Figure 7 shows an appreciable attenuation of the acceleration; meanwhile, it can be appreciated that the velocity curves have been smothered. Figure 6 suggests that, mainly, the filter at case 4 has a better performance in this matter. In Figure 8, the velocities and the accelerations concerning case 3 (randomly selected) of Table 1 are isolated in order to present a comparison to appreciate the differences between the filtering process with 120 and 150 data points.

Smoother signals are appreciated in the cases where 150 data points were available for the analysis, which, alongside the results presented in [13,14,15], led to the conclusion that the more data points available, the better the estimation process. Nevertheless, conclusions cannot be firmly established at this point of the data treatment process, as the Least Squares Method has not been used yet.

A second filter was applied to the already filtered accelerations obtained at cases 2–5, as suggested by [11,12,13]. For such a task, the second filter parameters, as shown in Table 2, were established, and new labels for these cases were assigned.

**Figure 9:**
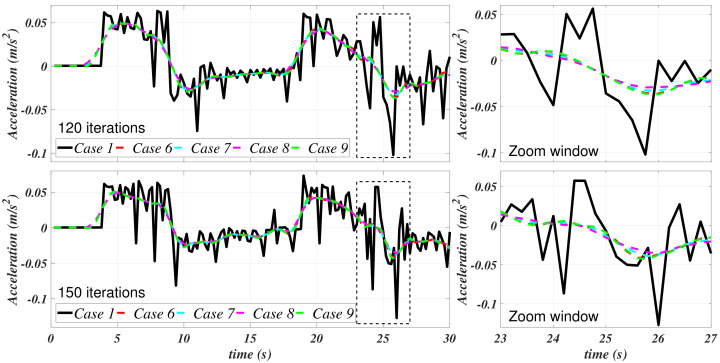
Double filtered acceleration signals in positive motion along ${}^{b}x$.

**Figure 10:**
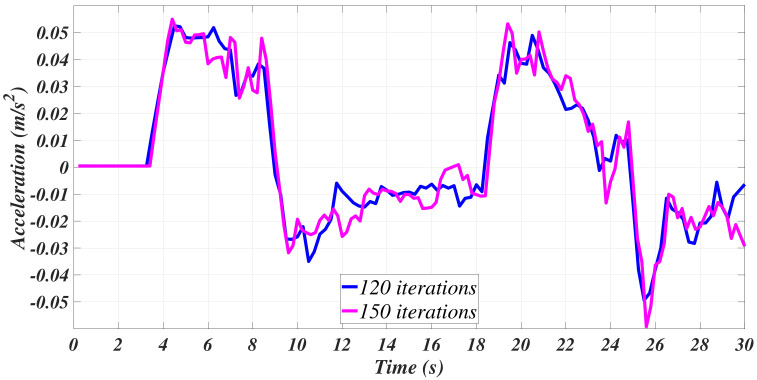
Comparative of the acceleration signals with double filter: case 8.

Figure 9 depicts the double-filtered accelerations, where the attenuation of the signals is notorious. Figure 10 shows case 8 of the data treatment strategy.

**Figure 11:**
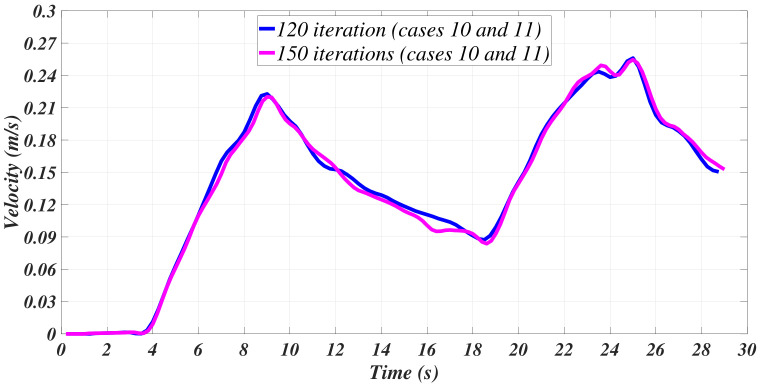
Filtered velocities with the Savitzky–Golay method.

**Figure 12:**
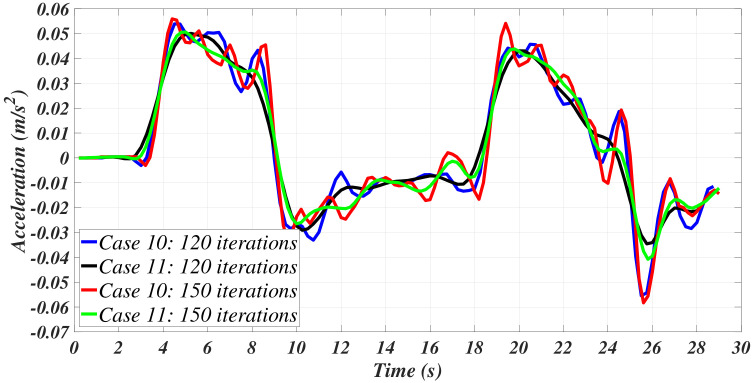
Filtered accelerations for cases 10 and 11.

Two supplementary cases were considered. Case 10 corresponds to the implementation of the sgolay function to filter the velocity and compute the acceleration from the velocity data provided. Finally, case 11 corresponds to case 10 yet, with an additional Savitzky–Golay filter applied to the acceleration signal that was previously computed by the sgolay function. Table 3 introduces the parameters for these two cases. Figure 11 and Figure 12 depict the corresponding results.

With the data acquired and arranged in the form of Equation (19), the expressions in Equations (16) and (17) were applied in order to obtain the estimated values of the hydrodynamic parameters.

In Appendix A, the tables containing the statistical data for each analysis are presented. Based on the data set that was obtained with 120 data points concerning the positive surge motion of the mini-AUV, the added mass value is observed to adopt a magnitude near 9kg, while the linear and quadratic damping coefficients are close to 0.1kg/s and 6kg/m, respectively.

By definition, the added mass and the damping matrices must be strictly positive definite, implying that $m_{x}>m$, $X_{u}>0$, and $X_{u\left|u\right|}>0$ [46,47]. Most of the parameters estimated with the 120 iterations simulation results do not satisfy the conditions mentioned above. On the other hand, on matters of the hydrodynamic coefficients that are computed with the data of the ANSYS CFX simulation with 150 iterations, it was found that more results fit the specifications, which suggests a close relationship between the accuracy of the estimation and the number of data points in such a manner that the more information is obtained, the more accurate the estimation may be. Several works in the literature have also given evidence of this result [8,9,10,37]. The fact that the standard deviation of the linear damping coefficient is close to the value of the parameter could be attributed to the data points used to compute the parameters [4,5,6,16,37].

Based on the lowest variance and standard deviation of the parameters in the Appendix A, and in comparison with the results exposed by [11,12,13,14], the estimation that is selected to be valid is that of the case 11; thus, the hydrodynamics coefficients that characterize the positive ${}^{b}x$ motion of the vehicle correspond to:

(41) $$X_{\dot{u}}=1.63082585\;kg,\;X_{u}=0.142306865\;kg/s,\;\mathrm{and}\;X_{u\left|u\right|}=5.153776644\;\mathrm{kg}/m$$

The same identification procedure was followed in order to estimate each of the hydrodynamic coefficients. The overall set of hydrodynamic parameters of the mini-AUV is available at Table 4.

Upon conclusion of the parameters identification procedure, the information obtained was used to define and simulate the controllers proposed in Section 4.

### 5.2. Control Simulations Results

**Figure 13:**
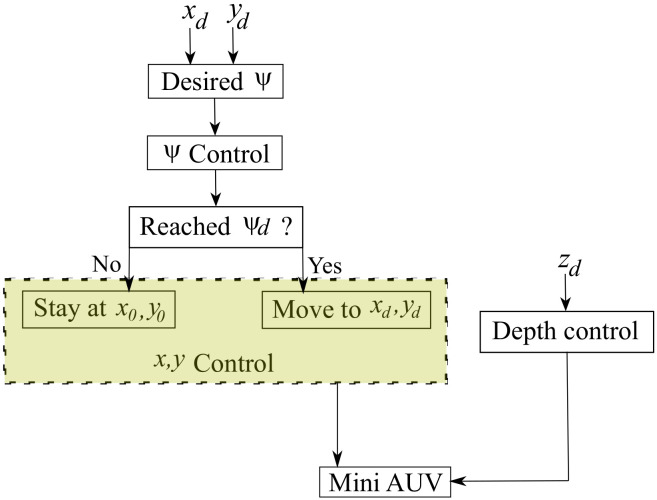
Control strategy for the mini-AUV.

Because the vertical motion of the mini-AUV is decoupled from the horizontal plane, the control algorithm is divided into two parts: one for the control depth and the other for the motion in the horizontal plane; as depicted by the block diagram in Figure 13. The motion of the mini-AUV in the ${}^{I}x$ and ${}^{I}y$ directions is coupled to the ψ dynamics. Subsequently, given a desired point in the space ${\left[x_{d}\;y_{d}\;z_{d}\right]}^{T}\in {I\;R}^{3}$, the desired angle $\psi_{d}$ is computed when considering the initial positions $x_{0}$ and $y_{0}$, such that:

(42) $$\psi_{d}=\tan^{-1}\left(\frac{y_{d}-y_{0}}{x_{d}-x_{0}}\right)$$

First, the mini-AUV is driven to the desired angle $\psi_{d}$ and, once it has reached the commanded set-point, it starts to move forwards to reach the desired point $x_{d}$, $y_{d}$. In the meantime, the depth (*z*) control drives the mini-AUV to the desired vertical position.

For the simulations, the geometric parameter $l_{y}$ was set to be 0.16 m, and the estimates of the hydrodynamic parameters, as introduced in Table 4, were used. Additionally, the gains of the PD, PID, and SM controllers correspond to those in Table 5. Such gains ensure a slow-motion of the vehicle, as it had been assumed in Section 2 and Section 4.

**Figure 14:**
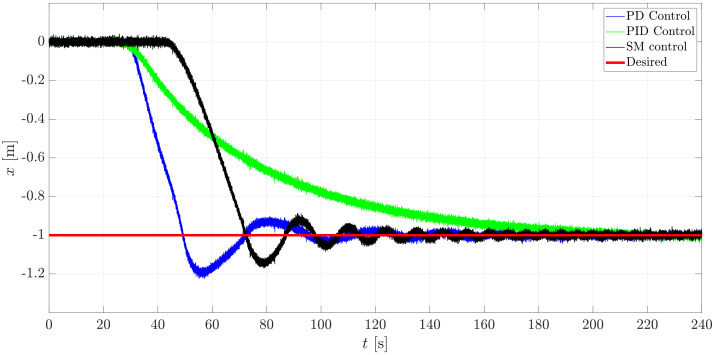
The translational behavior of the mini-AUV along ${}^{I}x$.

**Figure 15:**
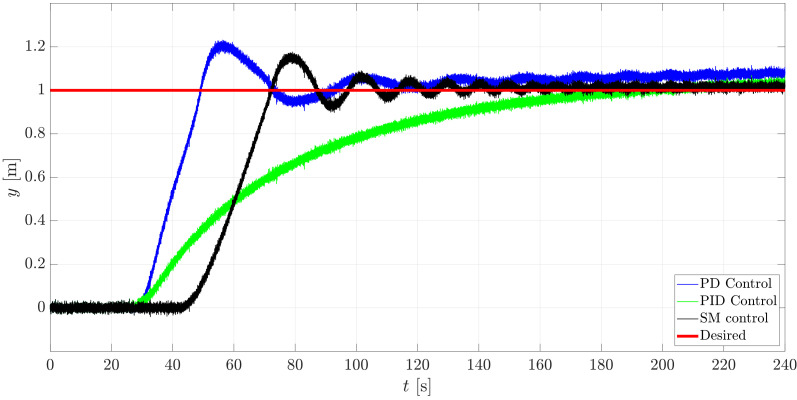
Translational behavior of the mini-AUV along ${}^{I}y$.

**Figure 16:**
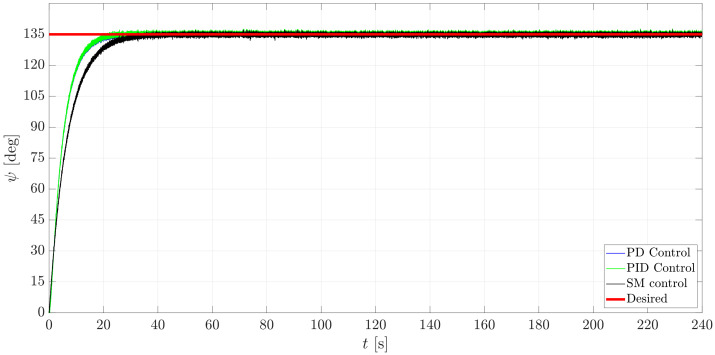
Rotational behavior of the mini-AUV about the axis ${}^{I}z$ (ψ motion).

**Figure 17:**
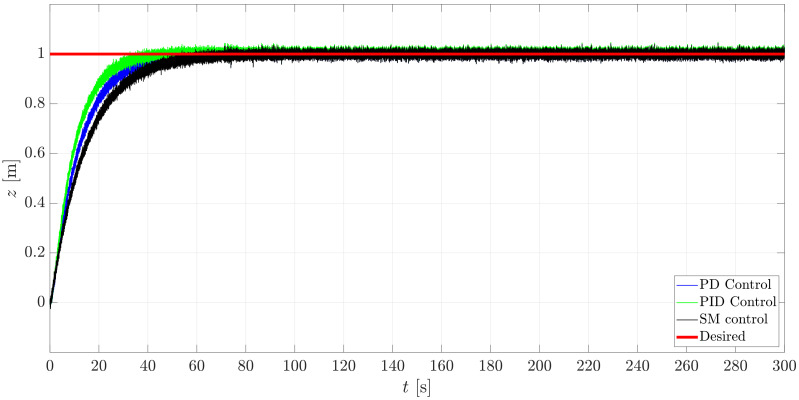
Translational behavior of the mini-AUV along ${}^{I}z$.

The simulation was run for a total time tsim=240 s with a constant sampling time dt=0.001 s in Matlab/Simulink™2018b while using a computer that was embedded with an 8 GB RAM and an Inter Core™i5-8250 CPU @ 1.60 GHz & 1.80 GHz processor. The initial conditions of the system were set to 0 and the reference point to reach was selected to be at $x_{d}=-1\;m$, $y_{d}=1\;m$, $z_{d}=1\;m$. The results of such control studies are exposed through Figure 14, Figure 15, Figure 16 and Figure 17.

In Figure 14 and Figure 15, it is possible to appreciate the performance of the controllers regarding the translational horizontal motion of the mini-AUV. In this matter, the difference in the settling time is notorious.

The mini-AUV under the PD and SM control techniques has an oscillatory behavior, while the system under the influence of the PID controller does not, due to the effect of the integral term. Nevertheless, such oscillatory behavior can be attenuated by a detailed tuning procedure, according to the desired performance or task requirements.

Figure 15 gives evidence of the steady-state response of the mini-AUV, which has a better performance when implementing the PID and SM controllers, since the error tends to 0 as times goes to infinity, which is produced due to the integral term in the case of the PID controller, and to the robust approach and conception of the Sliding Mode controller.

Based on the gains presented in Table 5, it can be observed that the PID and PD control drive the mini-AUV to $\psi_{d}$ at similar times; on the other hand, the SM controller drives the system to $\psi_{d}$ more slowly, yet, in the three cases, the desired orientation seemed to be reached, which is reinforced by the result that is depicted in Figure 16.

Concerning the ψ dynamics, no oscillation behavior was desired as an arbitrary requirement, thus the set of control gains previously introduced ensures such a requirement. Not a notorious difference is appreciated when the PD and PID controllers are applied, yet the response of the mini-AUV, when controlled by the SMC, is slower, which cannot be considered to be an issue, since it can be fixed by selecting a new set of gains.

In Figure 17, for the motion in ${}^{I}z$, a difference between the transient response of the three controllers is appreciable, yet, similar results can be obtained by selecting the proper set of parameters, depending on the controller.

Some considerations have to be taken, even when the three controllers accomplish the goal of controlling the position of the vehicle. The results suggest that these controllers can successfully drive the system to the desired point, yet external disturbances influences have not been considered; thus, this scenario must be validated in simulations and reality. Besides, it can be commented that a better performance is expected from the SM controller, since it is a robust control technique.

## 6. Concluding Remarks and Future Work

In this paper, the issue of identifying the hydrodynamic parameters of a mini-AUV in the early stages of the design was treated. In this regard, the dynamic model has been described and simplified in order to study the position stability problem under three different control approaches. The robot manipulator control theory was used to design the three controllers and study the stability of the closed-loop system. The adaptation mentioned above may be considered to be a new control design methodology for this kind of vehicle.

The hydrodynamic parameters of the mini-AUV were estimated by close-to-reality hydrodynamics simulations. A real experimentation procedure and the environmental conditions were adapted; additionally, the Immersed Solid option in ANSYS CFX to analyze the behavior of these vehicles may establish new options and capabilities to be explored and exploited in the upcoming works. Such options, alongside the recent founds and improving methods on the Least Squares estimation technique [20,49,53,54,55], could improve the estimation of such parameters and lead to more efficient simulation algorithms. Nevertheless, some issues that are described in [6] can emerge, thus a careful use of the CFD software is suggested, yet it is a useful tool in early design stages, the estimation of the parameters should take place in real and virtual scenarios.

A detailed and refined 3D model of the mini-AUV can be improved in the upcoming projects to increase the efficiency of the vehicle in operation, based on the results that were provided by the ANSYS CFX software, since the hydrodynamic parameters can be considered to be a tool for measuring the design efficiency [4,5,6,16]. A continuous improvement process also implies exploring more options and several parameters configuration to develop the simulations and obtain more accurate estimations, not to mention the real experimentation and problems that come with the data acquisition task.

The use of a Work Station Computer is strongly encouraged, since this would allow for performing faster simulations; thus, more data points can be obtained, and the precision of the estimation can be improved as well as the quality of the mesh. Performinf more simulations considering different real approaches and new methodologies is part of the upcoming work, as suggested by [4,5,6].

Numerical simulations validated the controllers; however, to control the mini-AUV, when considering the whole phenomena in its dynamics and uncertainties, is opened to new projects at the Institute. This manuscript pretends to serve as an introductory work to the conception procedure of underwater vehicles and their hydrodynamic parameters identification.

## Figures and Tables

**Table 1:** Parameters of the Savitzky–Golay filter.

Case	*u* Filter		$\dot{u}$ Filter	
	k	f	k	f
1	-	-	-	-
2	1	3	1	3
3	1	5	1	5
4	1	7	1	7
5	3	7	3	7

**Table 2:** Parameters of the second Savitzky–Golay acceleration filter.

Case	*u* Filter		$\dot{u}$ 1st Filter		$\dot{u}$ 2nd Filter	
	k	f	k	f	k	f
6	1	3	1	3	1	7
7	1	5	1	5	1	7
8	1	7	1	7	1	7
9	3	7	3	7	1	7

**Table 3:** Parameters of the Savitzky–Golay method.

Case	*u* Sgolay		$\dot{u}$ Savitzky–Golay Filter		
	N	F		k	f
10	4	11		-	-
11	4	11		1	7

**Table 4:** Selected estimations of the hydrodynamic parameters.

Parameter	For Positive	For Negative
	Velocity	Velocity
$X_{\dot{u}}$ $\left(\mathrm{kg}\right)$	1.630825	1.932838
$X_{u}$ $\left(\mathrm{kg}/s\right)$	0.142306	0.091036
$X_{u\left|u\right|}$ $\left(\mathrm{kg}/m\right)$	5.153776	5.868182
$Z_{\dot{w}}$ $\left(\mathrm{kg}\right)$	2.588308	3.908386
$Z_{w}$ $\left(\mathrm{kg}/s\right)$	7.157339	7.632676
$Z_{g}$ $\left(N\right)$	−14.309872	−14.309872
$N_{\dot{r}}$ $\left(\mathrm{kg}\cdot m^{2}\right)$	0.0240417	0.0240417
$N_{r}$ $\left(\mathrm{kg}\cdot m^{2}/s\right)$	0.013791	0.013791

**Table 5:** Controllers gains for the simulation of the mini-AUV.

PD	Value	PID	Value	SMC	Value
$k_{p_{x}}$ (kg/s${}^{2}$)	0.2	$k_{p_{x}}$ (kg/s${}^{2}$)	0.012	$K_{z_{x}}$ (m/s${}^{2}$)	0.05
$k_{p_{y}}$ (kg/s${}^{2}$)	0.2	$k_{p_{y}}$ (kg/s${}^{2}$)	0.012	$K_{z_{y}}$ (m/s${}^{2}$)	0.05
$k_{p_{z}}$ (kg/s${}^{2}$)	1	$k_{p_{z}}$ (kg/s${}^{2}$)	1	$K_{z_{z}}$ (m/s${}^{2}$)	1
$k_{p_{\psi }}$ (Nm)	0.1	$k_{p_{\psi }}$ (Nm)	0.01	$K_{\psi }$ (s${}^{-2}$)	1
$k_{v_{x}}$ (kg/s)	2	$k_{v_{x}}$ (kg/s)	0.005	$\lambda_{x}$ (s${}^{-1}$)	0.7
$k_{v_{y}}$ (kg/s)	2	$k_{v_{y}}$ (kg/s)	0.005	$\lambda_{y}$ (s${}^{-1}$)	0.7
$k_{v_{z}}$ (kg/s)	5	$k_{v_{z}}$ (kg/s)	3	$\lambda_{z}$ (s${}^{-1}$)	0.07
$k_{v_{\psi }}$	0.5	$k_{v_{\psi }}$	0.5	$\lambda_{\psi }$	0.15
		$k_{i_{x}}$ (kg/s${}^{2}$)	0.0003		
		$k_{i_{y}}$ (kg/s${}^{2}$)	0.0003		
		$k_{i_{z}}$ (kg/s${}^{2}$)	0.0005		
		$k_{i_{\psi }}$ (Nm)	0.00005		

## Data Availability

Simulation data are available on request to José J. Castillo-Zamora.

## Appendix A. Additional Data Obtained at the Hydrodynamics Parameters Estimation Process

Appendix A provides the statistical data obtained during the estimation of the hydrodynamic parameters exposed throughout the paper. Table A1 and Table A2 correspond to the analysis of the positive motion of the vehicle in ${}^{b}x$, and Table A3 stands for the negative motion in ${}^{b}x$. The results concerning the treatment of the data for the positive and negative motion of the vehicle in ${}^{b}z$ are exposed in Table A5 and Table A4, respectively. Lastly, Table A6 shows the parameters estimation process results regarding the rotation motion about ${}^{b}z$ (ψ). The data highlighted with bold stands for the estimated hydrodynamic parameters that were considered valid as they fulfill the requirements discussed in Section 5.

**Table A1:** Estimation of hydrodynamic parameters for the positive motion in ${}^{b}x$ with 120 iterations.

Case	1	2	3	4
$m_{x}\;\left(\mathrm{kg}\right)$	5.765440244	7.722155889	8.703062008	9.068444505
$X_{u}\;\left(\mathrm{kg}/s\right)$	0.394198185	0.087986129	−0.053646189	−0.119567686
$X_{u\left|u\right|}\;\left(\mathrm{kg}/m\right)$	4.102304926	5.591797761	6.326064633	6.70061939
Variance	0.018398838	0.009261478	0.00571343	0.006470697
Stnd Dev $m_{x}$	0.373086205	0.318102822	0.271556001	0.303454831
Stnd Dev $X_{u}$	0.346590739	0.248908439	0.19783262	0.212992615
Stnd Dev $X_{u\left|u\right|}$	1.782154508	1.282058946	1.022656101	1.106084553
**Case**	**5**	**6**	**7**	**8**
$m_{x}\;\left(\mathrm{kg}\right)$	7.64703005	9.21518067	9.385690628	9.566054714
$X_{u}\;\left(\mathrm{kg}/s\right)$	0.135204424	−0.052398199	−0.082941606	−0.123623731
$X_{u\left|u\right|}\;\left(\mathrm{kg}/m\right)$	5.361670536	6.225837822	6.430655651	6.684074805
Variance	0.008921403	0.006293474	0.00595281	0.006132446
Stnd Dev $m_{x}$	0.308064692	0.303419582	0.29964355	0.31056563
Stnd Dev $X_{u}$	0.243119075	0.205816942	0.202121283	0.207350628
Stnd Dev $X_{u\left|u\right|}$	1.250463857	1.059446073	1.044496664	1.076580065
**Case**	**9**	**10**	**11**	
$m_{x}\;\left(\mathrm{kg}\right)$	7.64703005	8.391070197	9.206834393	
$X_{u}\;\left(\mathrm{kg}/s\right)$	0.135204424	0.073448775	0.004971469	
$X_{u\left|u\right|}\;\left(\mathrm{kg}/m\right)$	5.361670536	5.665845761	5.944058517	
Variance	0.008921403	0.006211526	0.006128335	
Stnd Dev $m_{x}$	0.308064692	0.277198866	0.301857089	
Stnd Dev $X_{u}$	0.243119075	0.210389465	0.209432835	
Stnd Dev $X_{u\left|u\right|}$	1.250463857	1.071664569	1.06616972	

**Table A2:** Results of the estimation of hydrodynamic parameters for the positive motion in ${}^{b}x$ with 150 iterations.

Case	1	2	3	4
$m_{x}\;\left(\mathrm{kg}\right)$	5.117152947	7.637939366	8.401739147	8.810009611
$X_{u}\;\left(\mathrm{kg}/s\right)$	−0.688362153	−0.369721766	−0.266771427	−0.190308172
$X_{u\left|u\right|}\;\left(\mathrm{kg}/m\right)$	−2.615927203	−4.150283399	−4.677750899	−5.082018777
Variance	0.023813223	0.009599942	0.006820511	0.006529014
Stnd Dev $m_{x}$	0.363228922	0.286740198	0.258334352	0.264353731
Stnd Dev $X_{u}$	0.34730935	0.222358949	0.188752273	0.186091016
Stnd Dev $X_{u\left|u\right|}$	1.776793931	1.138640192	0.968753797	0.957926171
**Case**	**5**	**6**	**7**	**8**
$m_{x}\;\left(\mathrm{kg}\right)$	7.569930729	8.966171898	9.072344669	9.222521444
$X_{u}\;\left(\mathrm{kg}/s\right)$	−0.393675317	−0.256729862	−0.235866828	−0.186813331
$X_{u\left|u\right|}\;\left(\mathrm{kg}/m\right)$	−4.031800872	−4.672459037	−4.812534801	−5.084410331
Variance	0.009589836	0.006068915	0.005989863	0.006152794
Stnd Dev $m_{x}$	0.283968253	0.257984418	0.259231581	0.267621685
Stnd Dev $X_{u}$	0.221672771	0.177160111	0.176990795	0.180647372
Stnd Dev $X_{u\left|u\right|}$	1.134017318	0.906868497	0.908260782	0.929839948
**Case**	**9**	**10**	**11**	
$m_{x}\;\left(\mathrm{kg}\right)$	7.569930729	8.305017728	**9.13082585**	
$X_{u}\;\left(\mathrm{kg}/s\right)$	−0.393675317	−0.231001365	**−0.142306865**	
$X_{u\left|u\right|}\;\left(\mathrm{kg}/m\right)$	−4.031800872	−4.7674745	**−5.153776644**	
Variance	0.009589836	0.006973362	**0.005825496**	
Stnd Dev $m_{x}$	0.283968253	0.260816606	**0.259120036**	
Stnd Dev $X_{u}$	0.221672771	0.195171189	**0.178821518**	
Stnd Dev $X_{u\left|u\right|}$	1.134017318	0.989958155	**0.906535646**	

**Table A3:** Results of the estimation of hydrodynamic parameters for the negative motion in ${}^{b}x$ with 150 iterations.

Case	1	2	3	4
$m_{x}\;\left(\mathrm{kg}\right)$	4.490766485	7.476342027	8.518860195	9.237964526
$X_{u}\;\left(\mathrm{kg}/s\right)$	0.693665481	0.292592974	0.111440887	0.035208908
$X_{u\left|u\right|}\;\left(\mathrm{kg}/m\right)$	2.916991222	4.905349207	5.801569548	6.211827813
Variance	0.028267535	0.012738937	0.008886547	0.006633462
Stnd Dev $m_{x}$	0.37350401	0.3343355	0.304900289	0.279102261
Stnd Dev $X_{u}$	0.398094845	0.270160539	0.228000587	0.198653983
Stnd Dev $X_{u\left|u\right|}$	2.121582311	1.440839276	1.218244546	1.064598718
**Case**	**5**	**6**	**7**	**8**
$m_{x}\;\left(\mathrm{kg}\right)$	7.396818781	9.435143478	9.602678894	9.806286025
$X_{u}\;\left(\mathrm{kg}/s\right)$	0.290536196	0.049737835	−0.028148947	−0.013924468
$X_{u\left|u\right|}\;\left(\mathrm{kg}/m\right)$	4.901303762	6.069494533	6.449949306	6.422202462
Variance	0.01305013	0.006110739	0.006417925	0.006205586
Stnd Dev $m_{x}$	0.336010698	0.272154653	0.284726263	0.285326144
Stnd Dev $X_{u}$	0.272728409	0.187983118	0.194393045	0.192392557
Stnd Dev $X_{u\left|u\right|}$	1.452850112	1.001725205	1.037955359	1.030669723
**Case**	**9**	**10**	**11**	
$m_{x}\;\left(\mathrm{kg}\right)$	7.396818781	8.447696759	**9.432838931**	
$X_{u}\;\left(\mathrm{kg}/s\right)$	0.290536196	0.190128043	**0.091036771**	
$X_{u\left|u\right|}\;\left(\mathrm{kg}/m\right)$	4.901303762	5.400394316	**5.868182533**	
Variance	0.01305013	0.007716584	**0.006124236**	
Stnd Dev $m_{x}$	0.336010698	0.280816445	**0.274928891**	
Stnd Dev $X_{u}$	0.272728409	0.215873225	**0.192786902**	
Stnd Dev $X_{u\left|u\right|}$	1.452850112	1.140846127	**1.018376008**	

**Table A4:** Results of the estimation of hydrodynamic parameters for the negative motion in ${}^{b}z$ with 150 iterations.

Case	1	2	3	4
$m_{z}\;\left(\mathrm{kg}\right)$	2.292646188	5.696928677	8.155996872	9.469217358
$Z_{w}\;\left(\mathrm{kg}/s\right)$	6.823495905	7.115760391	7.339611974	7.476632539
$Z_{g}\;\left(N\right)$	−13.8553377	−14.0199111	−14.145331	−14.2203024
Variance	0.449265112	0.29264543	0.193421042	0.168973397
Stnd Dev $m_{z}$	0.377118115	0.495312033	0.493076209	0.516926018
Stnd Dev $Z_{w}$	0.279542905	0.228175279	0.18747121	0.176925072
Stnd Dev $Z_{g}$	0.138675692	0.113488356	0.093421496	0.0882791
**Case**	**5**	**6**	**7**	**8**
$m_{z}\;\left(\mathrm{kg}\right)$	5.938688654	10.10754985	10.76495451	**11.4083869**
$Z_{w}\;\left(\mathrm{kg}/s\right)$	7.130143256	7.454997333	7.543491172	**7.632676342**
$Z_{g}\;\left(N\right)$	−14.0309875	−14.2150142	−14.2626364	**−14.3098727**
Variance	0.274305565	0.149793176	0.131641757	**0.12178331**
Stnd Dev $m_{z}$	0.48346965	0.506240918	0.496024952	**0.499068899**
Stnd Dev $Z_{w}$	0.22076476	0.165605526	0.156247509	**0.151410383**
Stnd Dev $Z_{g}$	0.109874512	0.082761713	0.078125774	**0.075744148**
**Case**	**9**	**10**	**11**	
$m_{z}\;\left(\mathrm{kg}\right)$	5.938688654	7.039846207	9.477346809	
$Z_{w}\;\left(\mathrm{kg}/s\right)$	7.130143256	6.671642701	6.75833619	
$Z_{g}\;\left(N\right)$	−14.0309875	−13.7838234	−13.8376442	
Variance	0.274305565	0.326174877	0.280832183	
Stnd Dev $m_{z}$	0.48346965	0.631375399	0.732671098	
Stnd Dev $Z_{w}$	0.22076476	0.23520344	0.218829433	
Stnd Dev $Z_{g}$	0.109874512	0.117923137	0.109872371	

**Table A5:** Results of the estimation of hydrodynamic parameters for the positive motion in ${}^{b}z$ with 150 iterations.

Case	1	2	3	4
$m_{z}\;\left(\mathrm{kg}\right)$	1.051614679	4.752953968	6.587469056	8.306230779
$Z_{w}\;\left(\mathrm{kg}/s\right)$	5.293985579	6.066173258	6.397884543	6.798312912
$Z_{g}\;\left(N\right)$	−13.3074485	−12.9363122	−12.7760468	−12.5819159
Variance	0.324015369	0.220089	0.180641832	0.142403005
Stnd Dev $m_{z}$	0.314127547	0.530858752	0.578877071	0.584207504
Stnd Dev $Z_{w}$	0.362108568	0.312915507	0.289395855	0.264295339
Stnd Dev $Z_{g}$	0.158922394	0.138398903	0.128488759	0.117945516
**Case**	**5**	**6**	**7**	**8**
$m_{z}\;\left(\mathrm{kg}\right)$	4.990263133	8.906760485	9.787928045	**10.08830879**
$Z_{w}\;\left(\mathrm{kg}/s\right)$	6.094918316	6.850862097	6.994152502	**7.157339123**
$Z_{g}\;\left(N\right)$	−12.9242288	−12.5459698	−12.480379	**−14.309872**
Variance	0.213614138	0.137502744	0.117958461	**0.110153575**
Stnd Dev $m_{z}$	0.534406829	0.605189466	0.588705344	**0.578252497**
Stnd Dev $Z_{w}$	0.308440059	0.260545523	0.243063523	**0.23836489**
Stnd Dev $Z_{g}$	0.136373993	0.116724808	0.108895784	**0.107017636**
**Case**	**9**	**10**	**11**	
$m_{z}\;\left(\mathrm{kg}\right)$	4.990263133	5.767758094	8.199786253	
$Z_{w}\;\left(\mathrm{kg}/s\right)$	6.094918316	4.877488253	4.995036925	
$Z_{g}\;\left(N\right)$	−12.9242288	−13.4829903	−13.4206186	
Variance	0.213614138	0.291733449	0.262690659	
Stnd Dev $m_{z}$	0.534406829	0.821382471	0.976860702	
Stnd Dev $Z_{w}$	0.308440059	0.322705147	0.307549992	
Stnd Dev $Z_{g}$	0.136373993	0.142323817	0.13591481	

**Table A6:** Results of the estimation of hydrodynamic parameters for the motion in ψ with 150 iterations.

Case	1	2	3	4
$I_{zz}\;\left({\mathrm{kgm}}^{2}\right)$	0.09150314	0.154938563	0.205543484	0.233317348
$N_{r}\;\left(\mathrm{kg}/s\right)$	0.01451316	0.014305644	0.014146321	0.014072976
Variance	0.000415892	0.000331019	0.000266467	0.000238701
Stnd Dev $I_{zz}$	0.014166958	0.016301838	0.016890015	0.017264417
Stnd Dev $N_{r}$	0.007894602	0.007063666	0.006365152	0.006056496
**Case**	**5**	**6**	**7**	**8**
$I_{zz}\;\left({\mathrm{kgm}}^{2}\right)$	0.152784249	0.24495803	0.258931023	**0.274659776**
$N_{r}\;\left(\mathrm{kg}/s\right)$	0.014292885	0.01387251	0.013782836	**0.01379101**
Variance	0.000331061	0.000224127	0.000208268	**0.000192268**
Stnd Dev $I_{zz}$	0.016080323	0.017150475	0.017042359	**0.016952814**
Stnd Dev $N_{r}$	0.007051294	0.005810967	0.00562722	**0.005435597**
**Case**	**9**	**10**	**11**	
$I_{zz}\;\left({\mathrm{kgm}}^{2}\right)$	0.152784249	0.181382573	0.243559836	
$N_{r}\;\left(\mathrm{kg}/s\right)$	0.014292885	0.01718263	0.017010375	
Variance	0.000331061	0.000299337	0.000228551	
Stnd Dev $I_{zz}$	0.016080323	0.016875525	0.017346636	
Stnd Dev $N_{r}$	0.007051294	0.006720781	0.005872666	
